# Cytoplasmic DNA can be detected by RNA fluorescence *in situ* hybridization

**DOI:** 10.1093/nar/gkz645

**Published:** 2019-07-24

**Authors:** Eliraz Greenberg, Hodaya Hochberg-Laufer, Shalev Blanga, Noa Kinor, Yaron Shav-Tal

**Affiliations:** The Mina & Everard Goodman Faculty of Life Sciences & Institute of Nanotechnology, Bar-Ilan University, Ramat Gan 5290002, Israel

## Abstract

Fluorescence *in situ* hybridization (FISH) can be used for the intracellular detection of DNA or RNA molecules. The detection of DNA sequences by DNA FISH requires the denaturation of the DNA double helix to allow the hybridization of the fluorescent probe with DNA in a single stranded form. These hybridization conditions require high temperature and low pH that can damage RNA, and therefore RNA is not typically detectable by DNA FISH. In contrast, RNA FISH does not require a denaturation step since RNA is single stranded, and therefore DNA molecules are not detectable by RNA FISH. Hence, DNA FISH and RNA FISH are mutually exclusive. In this study, we show that plasmid DNA transiently transfected into cells is readily detectable in the cytoplasm by RNA FISH without need for denaturation, shortly after transfection and for several hours. The plasmids, however, are usually not detectable in the nucleus except when the plasmids are efficiently directed into the nucleus, which may imply a more open packaging state for DNA after transfection. This detection of plasmid DNA in the cytoplasm has implications for RNA FISH experiments and opens a window to study conditions when DNA is present in the cytoplasm.

## INTRODUCTION

Development of the *in situ* hybridization (ISH) technique by Joe Gall enabled the detection of specific DNA sequences using radioactive probes (1,2). Later on, the method was adapted for the detection of RNA (3). With the conjugation of fluorescent moieties to the probes used for hybridization, this method became widespread and is referred to as fluorescence *in situ* hybridization (FISH) (4,5). RNA FISH can provide the detection of RNA at the level of single molecules (6). The current single molecule FISH (smFISH) techniques use probe sets containing dozens of ∼20-mer single-stranded DNA probes that specifically hybridize with the transcripts to detect individual RNAs (7).

Probe hybridization to DNA or RNA sequences is based on Watson–Crick base pairing, and therefore binding must occur on single stranded DNA or RNA. Therefore, DNA FISH protocols require a denaturation step that opens the double-stranded DNA. Denaturation is obtained by incubation of the fixed cells at high temperature and at a low pH. These conditions are considered harsh and cause the destruction of the RNA.

RNA FISH does not provide conditions for the detection of DNA since there is no need for a denaturation step with single stranded RNA. Secondary structures in the RNA are relaxed by incubation with formamide. Therefore, double-stranded genomic DNA in the nucleus is not detected by RNA FISH. Combining of RNA and DNA FISH within one experiment requires specially optimized protocols (8–10). The typical RNA smFISH protocol is known not to accommodate DNA detection, not only because the DNA remains inaccessible, but since the Tm of the short single-stranded probes used, is lower in DNA than in RNA, and would therefore be competed away by the complementary DNA strand (see Stellaris^®^ website).

We were therefore surprised to find that probe sets consisting of short single-stranded probes that were designed for the detection of RNAs by RNA FISH, could detect plasmid DNA in the cytoplasm following transient transfection, without a denaturation step. The detection of DNA was verified by different means including the use of plasmids containing gene sequences that cannot transcribe. The DNA was prominently observed in many cytoplasmic puncta, whereas in the nucleus there was usually no obvious detection. This raises implications regarding the packaging of DNA in the cytoplasm and the nucleus and the ability to detect DNA in the cytoplasm.

## MATERIALS AND METHODS

### Plasmids

The plasmids containing the MEG3 gene (Homo sapiens NR_033358) and the human PANDA sequence including the CDKN1A promoter region (human chromosome 6: 36,672,641–36,676,459) were synthesized by Rhenium (Israel). The PANDA plasmid used in the study did not contain the promoter region (except for the experiments in Supplementary Figure S1) and was generated by amplifying the PANDA sequence from the plasmid described above using PCR with primers that contain the appropriate restriction sites, and sub-cloned into pUC19 plasmid:

Forward: 5′-ATAGGTACCACGAATTCTTTCAGGAATGCC.

Reverse: 5′-ATAAAGCTTGCAGTGGCTCACGCCTGTAAT.

The removal of the ori region from this plasmid in order to create an ori-less plasmid was performed by the restriction enzymes ScaI and HindIII. This plasmid was transfected as a linear plasmid. The FRT plasmid containing the cyclin D1 coding region and 24xMS2 repeats in the 3′UTR of cyclin D1, did not contain a promoter region and was generated in (11). The FRT plasmid containing the PANDA and the cyclin D1 + MS2 sequences on different strands was generated as follows. First, the PANDA sequence including the promoter was amplified by PCR from the plasmid described above, using primers that contain the appropriate restriction sites and was then sub-cloned into the pUC19 plasmid:

Forward: 5′-ATAAAGCTTGCAGTGGCTCACGCCTGTAAT.

Reverse: 5′-ATAACCGGTCACAAGCACACATGCATCAGA.

Also, 24xPP7 sequences were removed from the plasmid CFP-24xPP7 (Addgene #40652) and sub-cloned into the pUC19-PANDA plasmid, and this whole region was cloned into the FRT plasmid with the cyclin D1 + MS2 sequences and no promoter, described above.

The plasmid containing the E6 gene was generated in (12) and is a Tet-inducible plasmid that is activated by the addition of doxycycline (dox) to the medium, and transcribes from a β-globin mini-gene fused to a CFP coding region with a SKL tri-peptide peroxisomal localization signal, and 18xMS2 repeats in the 3′UTR. The plasmid containing MS2 repeats only (pSL-24xMS2, Addgene #31865) does not contain a promoter (13).

### Cell culture

Human U2OS and U2OS Tet-On cells were maintained in low glucose Dulbecco's modified Eagle's medium (DMEM, Biological Industries, Israel) containing 10% fetal bovine serum (FBS, HyClone). HeLa and NIH3T3 cells were maintained in high glucose DMEM containing 10% FBS. U2OS cells stably expressing the E6 inducible gene (12) were transcriptionally induced with 1 μg/ml doxycycline overnight. Plasmids were transfected overnight with the following transfection reagents: PolyJet (SignaGen Laboratories), Lipofectamine 2000 (Thermo Fisher Scientific), FuGENE-HD (Promega) and Avalanche-Omni (EZ Biosystems). PolyJet was used in most of the experiments unless noted otherwise. Cells were also transfected overnight with calcium phosphate or allowed to express for 6 hrs after nucleofection (Amaxa). Actinomycin D (Sigma) was used for transcriptional inhibition (5 μg/ml, 2 h). Arsenite (Sigma) was added to the medium for stress granule formation (1 mM, 45 min) (14). For amino acid starvation, cells were washed twice with 1xPBS and incubated with EBSS media (Biological Industries) for 8 hrs to increase P body (PB) numbers (15). For RNA digestion, cells were grown on coverslips and treated with ActD for 2 hrs. Then, cells were permeabilized in 0.5% Tx-100 in PBS for 2 min and digested with RNase A (100 mg/ml in PBS with 3 mM MgCl_2_, Sigma) for 45 min at room temperature and then fixed in 4% paraformaldehyde (PFA) for 20 min. For DNase I (Sigma) treatment, cells were first fixed in ice cold methanol for 2 min, and then incubated (100 mg/ml, 5 mM MgCl_2_) for 2 h at room temperature.

### Fluorescence *in situ* hybridization

RNA FISH experiments with Stellaris (Biosearch Technologies) probes were performed according to the manufacturer's adherent cell protocol. Cells were seeded on coverslips a day before fixation. Cells were fixed in 4% PFA for 20 min, washed in PBS, and then incubated in 70% ethanol at 4°C overnight. Cells permeabilized in ethanol can be stored up to a week before hybridization. On the next day, the cells were incubated for 5 min at room temperature in wash buffer (10% formamide in 2× saline sodium citrate (SSC)). Then, the coverslips were transferred face down onto a drop of 100 μl of hybridization buffer (10% formamide, 2× SSC and 10% Dextran sulfate) containing 125 nM probe, in a humidified chamber sealed with parafilm and incubated in the dark at 37°C overnight. The humidified chamber prevents evaporation of the probe solution. Hybridization buffer should be prepared fresh and it is recommended to make excess volume of buffer due to viscosity of the solution. The hybridization step can be performed for shorter times dependent on several factors such as the hybridization strength of the probe set to the desired mRNA and the expression level of the transcript examined. After hybridization, the coverslips were transferred face up to a fresh well and washed twice in wash buffer in the dark at 37°C, for 30 min each wash, and then washed briefly in 1× PBS. Hoechst was used as a DNA counterstain. Cells were mounted in p-phenylenediamine mounting medium. Probes used were generated by Stellaris: Cy3-labeled PANDA probes (SMF-1063), Cy3-labeled human CCND1 probes (VSMF-2046), Cy5-labeled CFP probes (16), Cy3-labeled human MEG3 (VSMF-20346). For poly(A)+ detection a Cy5-labeled oligo-dT probe was used (14), and for MS2 repeats detection a Cy5-labeled probe was used: CTAGGCAATTAGGTACCTTAGGATCTAATGAACCCGGGAATACTGCAGAC.

The probe for DNA FISH was amplified by PCR from the plasmid containing the PANDA gene using the primers: Forward- 5′ GCCTGTTCCTCAATCCAAGA. Reverse- 5′ TTTGCTTCTGGGCAGAACTT. The PCR product (150 bp) was labeled with Green 496 dUTP using a Nick Translation DNA Labeling System 2.0 (Enzo) and precipitated in 0.3 M sodium acetate pH 5.2, 2.5× volume of 100% ice cold ethanol, 3 μg Cot1 DNA, at –70°C for 15 min. DNA was pelleted via centrifugation at 12 000 rpm for 30 min at 4°C. After air-drying, the pellet was resuspended in nuclease-free water. Cells were seeded on chamber slides (ibidi) and transfected with the PANDA plasmid by PolyJet, Lipofectamine 2000 or FuGENE-HD. One day after transfection, the cells were fixed by three 5 min washes in methanol:acetic acid (3:1). The slide was dried at room temperature (RT) for overnight. The probe was diluted in hybridization buffer (50% formamide, 10% dextran sulfate, 2% SSC and 1% Tween20). DNA on the slides was denaturated with the probe solution for 3 min at 75°C, and then the slide was incubated in a humidity chamber at 37°C for overnight. Washes after hybridization were performed in 0.4× SSC for 5 min at 72–74°C and then in 4× SSC with 0.1% Tween20 for 2 min at RT. Cells were washed briefly with DDW, and Hoechst was used as a DNA counterstain.

### Immunofluorescence

Cells were grown on coverslips, washed with PBS and fixed for 20 min in 4% PFA. Cells were then permeabilized in 0.5% Triton X-100 for 2.5 min. Cells were washed twice with PBS and blocked with 5% BSA for 20 min and immunostained for 1 h with a primary antibody. After three washes with PBS, the cells were incubated for 1 h with secondary fluorescent antibodies. Primary antibodies: mouse anti-G3BP1 (Abcam) and mouse anti-Hedls (Santa Cruz). Secondary antibodies: Alexa488-labeled goat anti-mouse IgG (Abcam). Nuclei were counterstained with Hoechst 33342 (Sigma) and coverslips were mounted in mounting medium.

### Fluorescence microscopy

Wide-field fluorescence images were obtained using the Cell^R^ system based on an Olympus IX81 fully motorized inverted microscope (60× PlanApo objective, 1.42 NA) fitted with an Orca-AG CCD camera (Hamamatsu) driven by the Cell^R^ software.

### Real time RT-PCR (qRT-PCR)

Total RNA was produced using Tri-Reagent (Sigma) and DNA was removed using the Turbo-DNase free kit (Ambion). Efficiency of DNA removal was examined by RT-PCR on the RNA sample after the DNase digestion. To achieve efficient DNase digestion, the RNA sample was diluted to 10 μg RNA in 50 μl reaction solution containing 1× Turbo-DNase buffer and 2 U Turbo-DNase at 37°C for 30 min. Higher concentrations of DNase (4–6 U) were added when the sample could not be diluted. To increase efficiency, the DNase reaction was performed in two steps by adding half of the Turbo-DNase to the reaction initially, incubation for 30 min, and then addition of the remainder of the enzyme for another 30 min. After DNase treatment, DNase inactivation reagent was added (0.1 volume) and mixed well. If >2 U of Turbo-DNase were used then inactivation buffer was doubled. The DNase-treated RNA was then incubated 5 min at room temperature and mixed occasionally. Then the RNA was centrifuged at 10 000× g for 1.5 min the supernatant containing the RNA was transferred to a fresh tube.

The cDNA (1 μg of RNA) was synthesized using the qScript cDNA Synthesis Kit (Quanta Biosciences). The real time qRT-PCR reaction contained 10 μl PerfeCTa SYBR Green FastMix (Quanta Biosciences), 0.6 μl of each primer (10 μM), 5 μl of diluted cDNA (25 ng) and 3.8 μl sterile water. Each sample was analyzed in triplicate. The qRT-PCR reaction was performed with the primers listed below on a CFX-96 system (Bio-Rad). The following cycling parameters were used: 95°C for 15 s for activation, followed by 39 cycles of denaturation at 95°C for 15 s, and annealing at 55°C for 30 s. Melting curve analysis was used to identify specific products increasing in 0.5°C increments every 5 s from 60 to 95°C. Every experiment was repeated three times. Analysis was performed with the software Bio-Rad CFX manager. Relative levels of PANDA mRNA were measured and normalized to the mRNA levels of ACTB, Tubulin and 18S rRNA. Primers used:

PANDA: Forward - GGGGCTGCCTATGTAGTGAA, Reverse - CCAGGTCTTGGATTGAGGAA.

ACTB: Forward - GCACAGAGCCTCGCCTT, Reverse - CCTTGCACATGCCGGAG.

Tubulin: Forward - GCCTGGACCACAAGTTTGAC, Reverse - TGAAATTCTGGGAGCATGAC.

18S rRNA: Forward - TGTGCCGCTAGAGGTGAAATT, Reverse - TGGCAAATGCTTTCGCTTT.

### Statistical analysis

The numbers of cytoplasmic puncta at different time points after transfection and under different treatments were compared using a one-way ANOVA, followed by a Tukey post hoc analysis. Homogeneity of variances and normality of residuals assumptions were checked graphically and statistically. The relative levels of PANDA mRNA were compared using a two-tailed *t* test.

## RESULTS AND DISCUSSION

### Plasmid DNA is detected in the cytoplasm by an RNA FISH protocol

**Figure 1.**
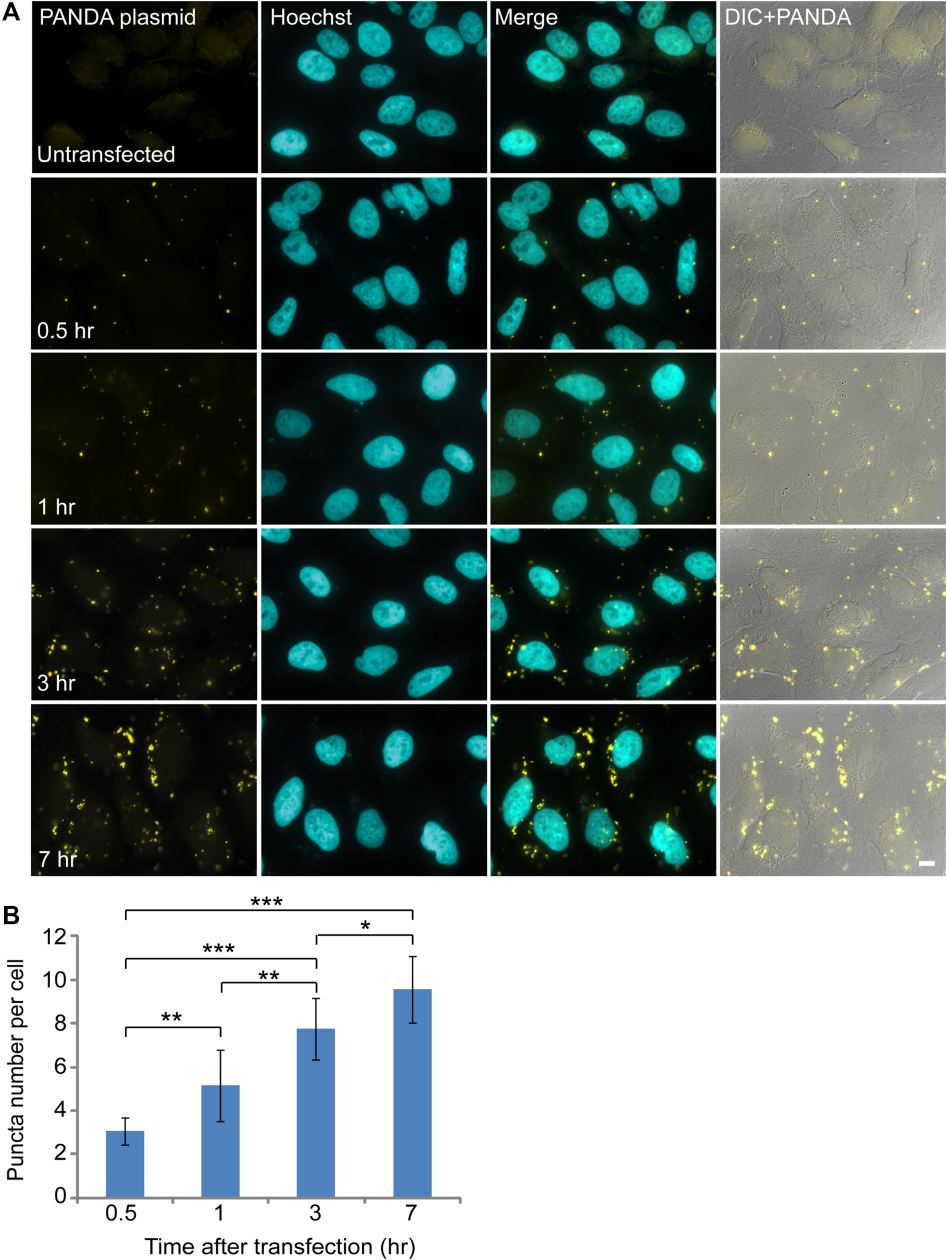
RNA FISH with probes to PANDA lncRNA shows an increase in the number and intensity of the cytoplasmic puncta over time after transfection. (**A**) U2OS cells were transiently transfected by PolyJet with a construct that contains the sequence of the PANDA lncRNA gene without a promoter. RNA FISH was performed with probes to the PANDA sequence. An increase in the number and intensity of the cytoplasmic puncta (yellow) was detected with the FISH probes at different time points after transfection (0.5, 1, 3 and 7 hrs). Hoechst DNA counterstain is in cyan. DIC is in gray. Scale bar = 10 μm. (**B**) The average numbers of cytoplasmic puncta are presented (three independent experiments; mean ± SD). There were significant differences in the numbers of the puncta counted between all the time points: between the time 0.5 h (*n* = 84) and the time points 1 h (*n* = 60), 3 h (*n* = 98), 7 h (*n* = 64) the *P*-values were *P* = 0.005, *P*< 0.001 and *P*< 0.001 respectively. Between the time 3 h and the time points 1 h, 7 h the *P*-values were *P*< 0.001 and *P* = 0.022. Between 7 h and 1 h, the *P*-value was *P*< 0.001. ***P < 0.001, **P < 0.01, *P < 0.05.

We were interested in detecting a long non-coding RNA (lncRNA) called PANDA (17) in human U2OS cells. The probe set we ordered for RNA smFISH did not show any detectable RNA signal. Since it was possible that the endogenous levels of PANDA were low, and to confirm that the probe set detects PANDA RNA, we transfected the cells with a plasmid that contained the genomic sequence that transcribes PANDA (from chr 6 in humans) including the promoter region. Subsequently, fluorescent puncta were observed, showing that the probe set was able to detect the PANDA sequence, however, the signal was localized prominently in many cytoplasmic puncta (Supplementary Figure S1A), providing a signal that was not reminiscent of RNA detected by RNA FISH that usually appear as very small dots. We then removed the promoter region and used a PANDA plasmid without a promoter (to be used hereon) that should not express this RNA. The cytoplasmic dots continued to appear (Figure 1A). Specifically, these puncta were not observed in untransfected cells, they appeared shortly after transfection, and their numbers and size increased in time after transfection supposedly due to the increased accumulation of plasmids entering the cells over time following transfection (Figure 1B).

**Figure 2.**
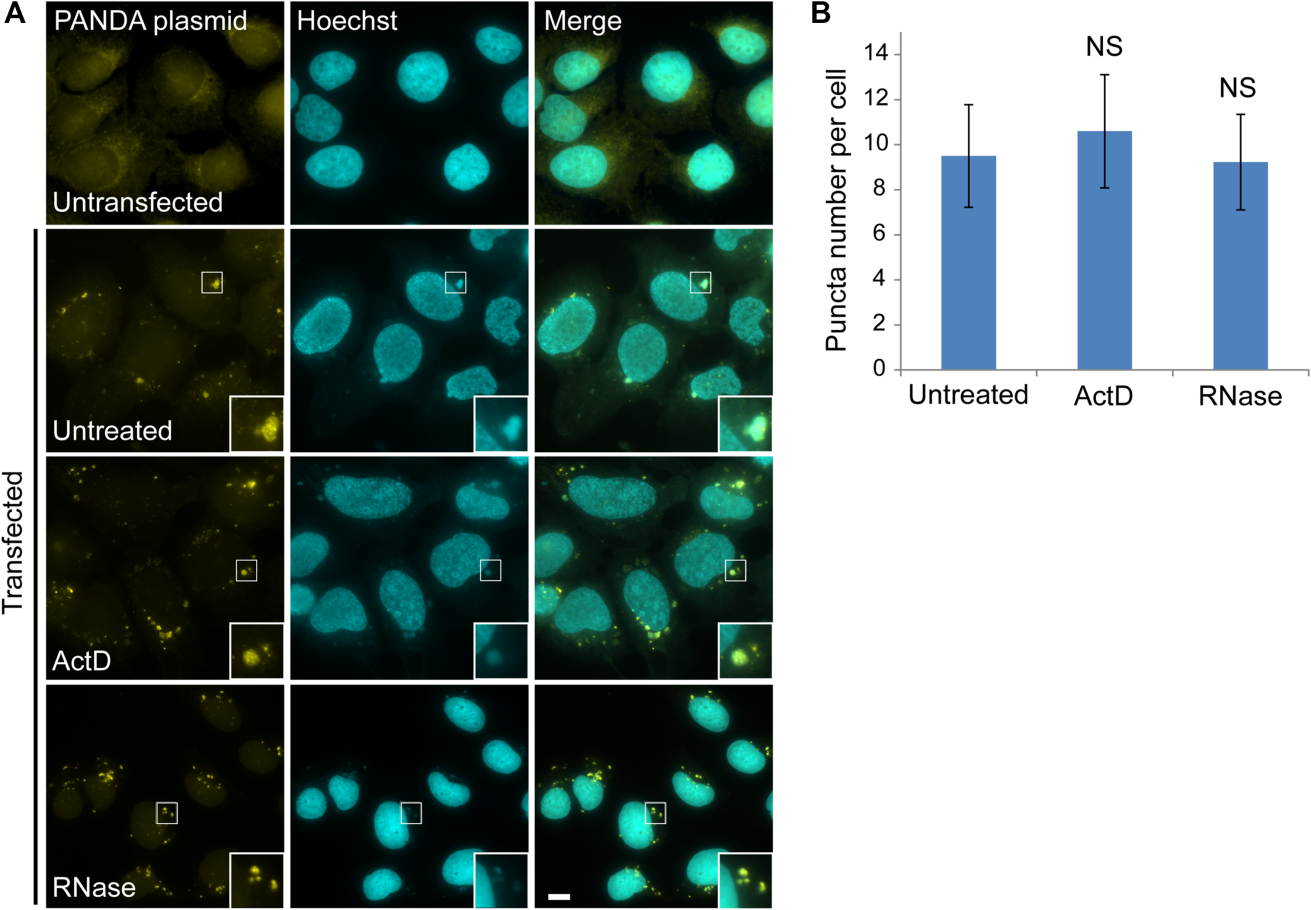
PANDA plasmid can be detected by RNA FISH. (**A**) U2OS cells were transiently transfected overnight by PolyJet with a construct that contains the PANDA gene (without a promoter). Untreated cells and cells treated with actinomycin D (2 hrs) or RNase all showed cytoplasmic puncta with a FISH probe set to PANDA (yellow) compared to untransfected cells. Enlarged areas in the images are shown in boxes. Hoechst DNA counterstain is in cyan. Scale bar = 10 μm. (**B**) The average numbers of cytoplasmic puncta counted are presented (mean ± SD). There were no significant variances between the numbers of puncta in untreated cells (*n* = 48) compared to cells treated with ActD (*P* = 0.066, *n* = 42) and RNase (*P* = 0.8303, *n* = 45). NS represents no significance.

We suspected that these puncta did not represent RNA. To examine this, the cells were treated with high levels of actinomycin D (ActD; 5 μg/ml) to inhibit transcription of all eukaryotic RNA polymerases, yet, the intensity of the cytoplasmic puncta was not diminished (Figure 2A middle, Supplementary Figure S1B). Next, the cells were treated with an RNase, and the cytoplasmic puncta persisted (Figure 2A bottom, Supplementary Figure S1C). There was no significant variance between the numbers of the cytoplasmic dots under the different treatments (Figure 2B). To verify that the cytoplasmic puncta were not known RNA-protein granules, we performed immunofluorescence with markers to such granules. The cytoplasmic dots appearing after transfection did not colocalize with cytoplasmic bodies containing RNAs such as P bodies or stress granules (18) (Supplementary Figure S2). Altogether, these experiments implied that cytoplasmic plasmid DNA was being detected by cognate RNA FISH probes even though the DNA was not denatured in the RNA FISH protocol.

### Plasmid DNA that cannot express RNA is detected in the cytoplasm after transient transfection

**Figure 3.**
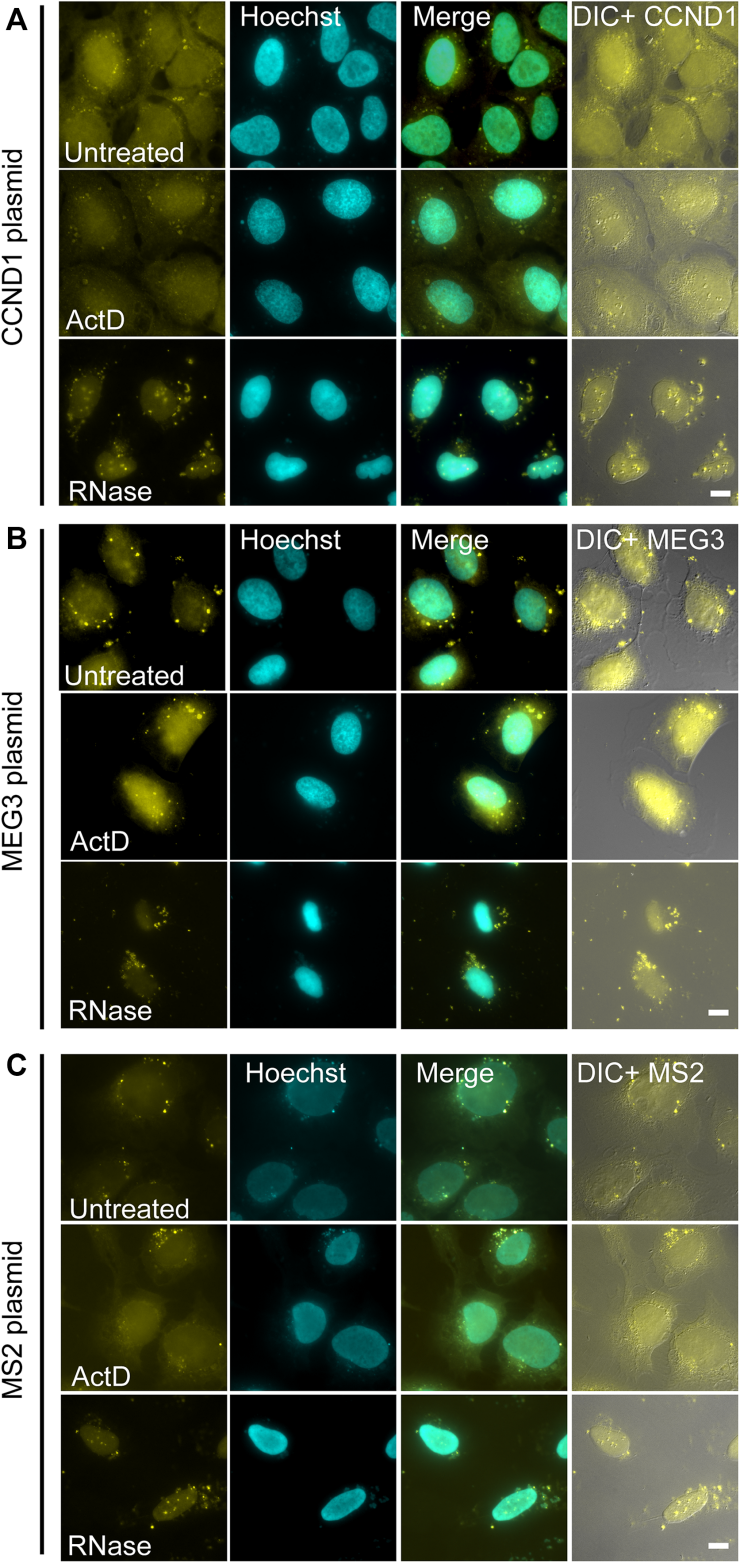
Different plasmids containing non-expressed genes are also detected in cytoplasmic puncta after transient transfection. U2OS Cells were transiently transfected overnight by PolyJet with constructs containing (**A**) the cyclin D1 (*CCND1*) gene without a promoter, (**B**) the sequence of the MEG3 lncRNA gene, without a promoter, and (**C**) only MS2 sequences without a promoter or a coding region. In all RNA FISH experiments the cells showed cytoplasmic puncta (yellow) with specific probe sets to each gene, and were still detected after actinomycin D (middle) or RNase (bottom) treatments. Hoechst DNA counterstain is in cyan. DIC is in grey. Scale bar = 10 μm.

To examine if the above detection of plasmid DNA in the cytoplasm by RNA FISH probes is a general phenomenon occurring when the RNA FISH protocol is applied to cells that were transiently transfected with plasmids, the analysis was expanded to other plasmids, cell types and transfection reagents. First, U2OS cells were transfected with three different plasmids that do not express mRNA (Figure 3): (i) A plasmid containing the cyclin D1 (CCND1) coding region and MS2 sequence repeats in the 3′UTR, but with no promoter (11); (ii) A plasmid containing the DNA sequence of the lncRNA MEG3 with MS2 sequence repeats in the 3′UTR, with no promoter; (iii) A plasmid with no promoter and no coding sequence containing the MS2 sequences only. RNA FISH experiments using specific probe sets for each gene showed cytoplasmic dots. These were observed also after ActD and RNase treatments (Figure 3).

The appearance of the cytoplasmic DNA dots was observed in other cell types we tested, such as human HeLa cells and mouse NIH3T3 cells (Supplementary Figure S3). In addition to transfection by the PolyJet transfection reagent used above, different transfection reagents were used: Fugene-HD a non-liposomal formulation, Lipofectamine consisting of cationic lipids, Avalanche-Omni a proprietary formulation of lipids and polymers, and all gave similar results (Supplementary Figure S4). An alternative non-liposomic protocol for transfection such as calcium phosphate, produced similar cytoplasmic puncta. Finally, nucleofection, an electroporation-based transfection technique that efficiently directs the insertion of plasmid DNA into the nucleus, showed the fluorescent dots in the nucleus 6 h after transcfection, meaning that the plasmid DNA can also be detected in the nucleus.

**Figure 4.**
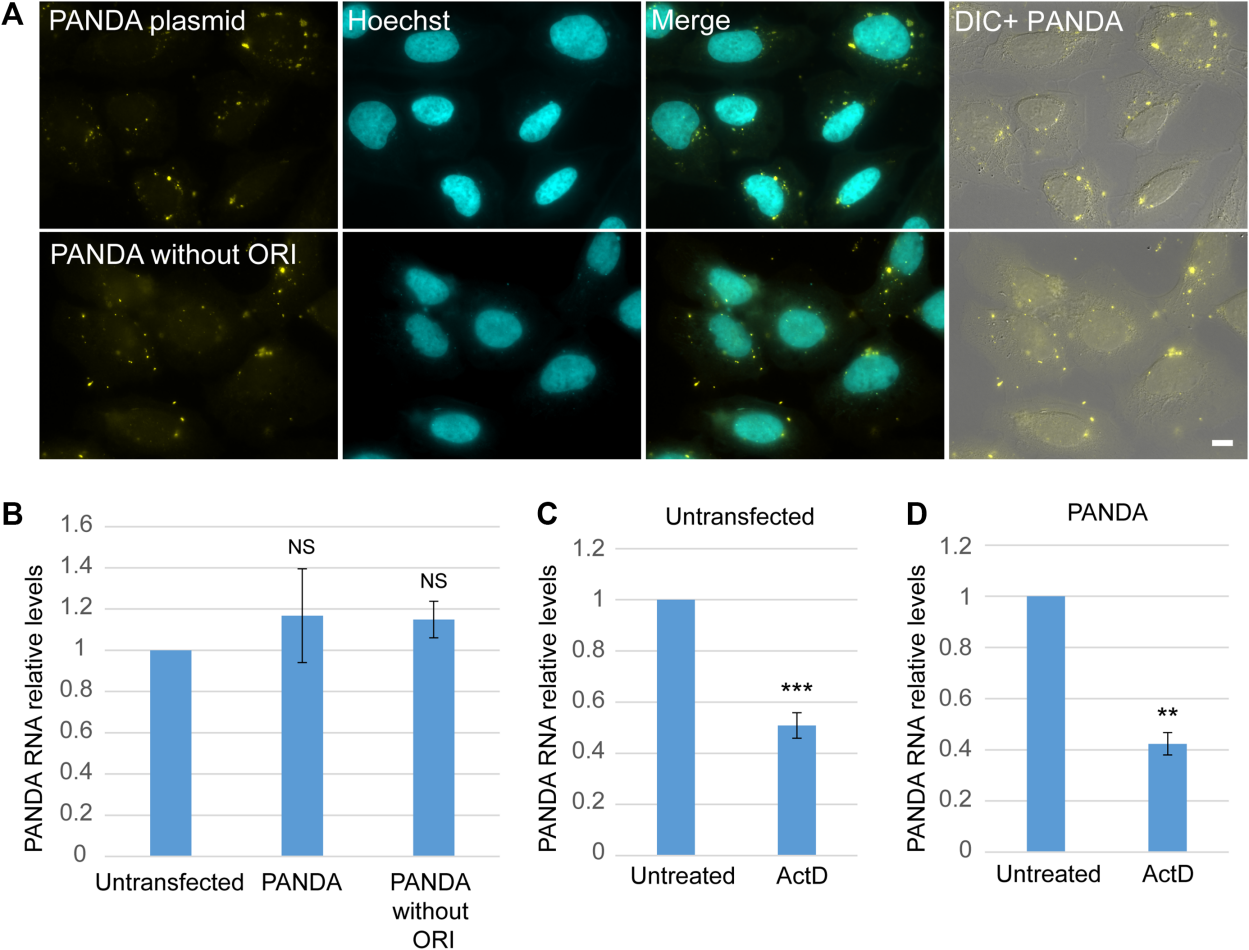
The PANDA plasmid does not have cryptic transcriptional activity. (**A**) U2OS cells were transiently transfected overnight by PolyJet with a construct that contains the PANDA gene without the promoter and without the ori region. The two plasmids showed cytoplasmic puncta with a FISH probe set to PANDA (yellow). Hoechst DNA counterstain is in cyan. DIC is in gray. Scale bar = 10 μm. (**B**) Real time qRT-PCR analysis of PANDA RNA levels in U2OS untransfected cells and in cells transfected with PANDA plasmids with or without the ori region. The average quantification of 3 repeated experiments is presented in the plots (mean ± sd). A two-tailed *t* test was performed. NS represents no significance. (**C**) qRT-PCR analysis of PANDA RNA levels in untransfected cells and (**D**) cells transfected with the PANDA plasmid under ActD treatment (2 h) and under normal conditions. The average quantification of three repeated experiments is presented in the plots (mean ± sd). A two-tailed *t* test was performed. ***P < 0.001, **P < 0.01.

To validate that the PANDA plasmid does not contain any cryptic internal transcriptional activity within the sequence or within the ori region (19), we also transfected an ori-less PANDA plasmid that was linearized by restriction enzymes to remove the ori region. This plasmid also formed cytoplasmic puncta (Figure 4A). We then measured the relative levels of PANDA RNA by qRT-PCR. This showed that the RNA levels were not increased in cells transfected with these two PANDA plasmids, without a promoter and without an ori, compared to untransfected cells (Figure 4B). The relative levels of PANDA RNA measured by qRT-PCR experiments represent the levels of the endogenous RNA since they were decreased under actinomycin D treatment both in untrasfected cells and in cells transfected with the PANDA plasmid (Figure 4C, D).

### RNA FISH can detect nuclear mRNA and cytoplasmic plasmid DNA

**Figure 5.**
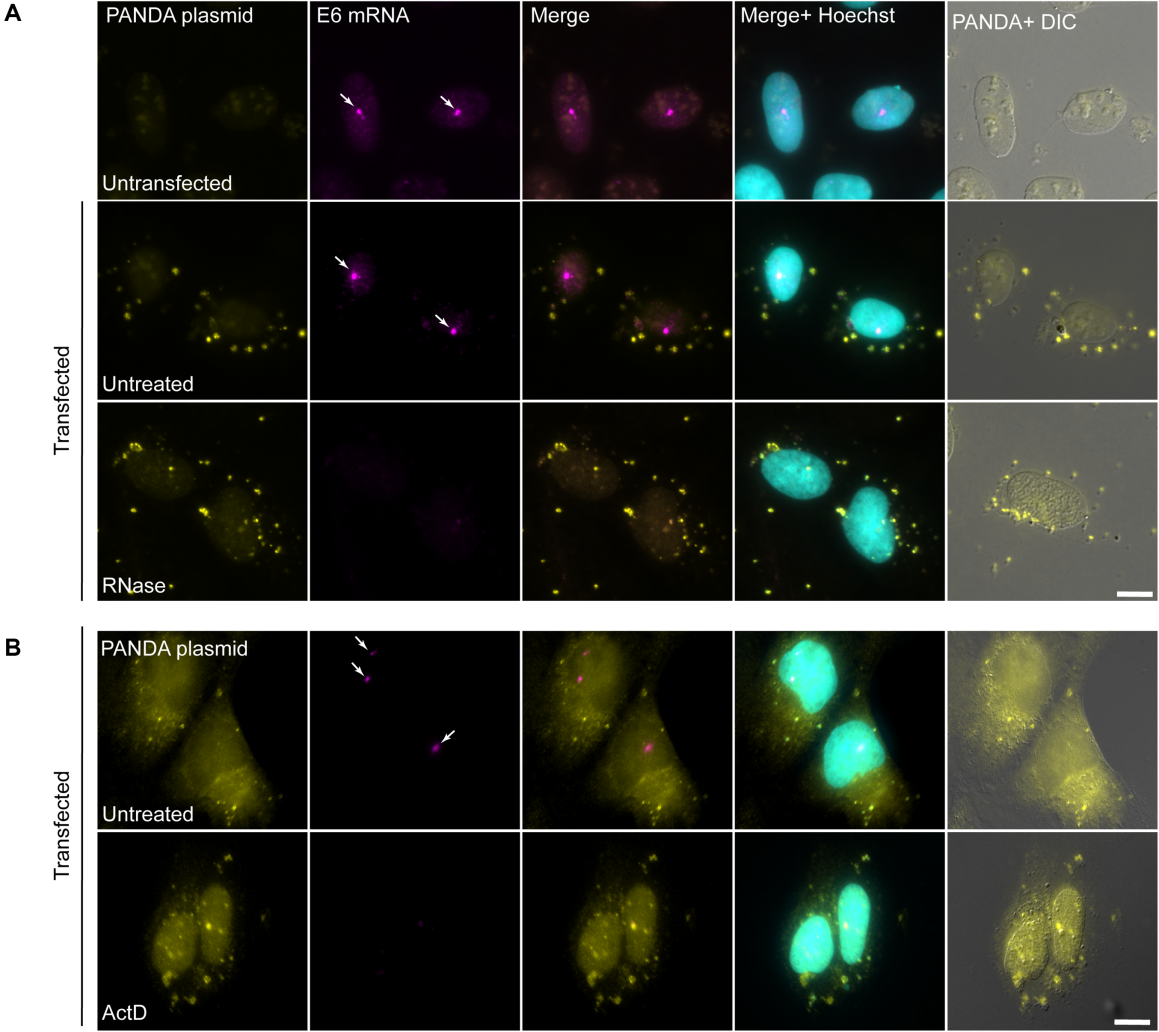
The PANDA FISH signal is not RNA. (**A**) U2OS cells stably containing the inducible E6 gene were transiently transfected overnight with the PANDA plasmid. RNA FISH was performed with PANDA probes (cytoplasmic dots, yellow) and probes that detect the CFP region in the mRNA transcribed from the E6 gene (nuclear transcription sites, magenta, arrows). Untreated cells showed both signals (middle), while the signal of the transcriptionally active E6 transcription sites was abolished under RNase treatment (bottom). (**B**) Similarly, actinomycin D transcription inhibition abolished the active transcription sites (arrows). Hoechst DNA counterstain is in cyan. DIC is in gray. Scale bar = 10 μm.

The above experiments suggested that the signal detected by the RNA FISH probes was DNA. To verify that the RNase was indeed removing RNA from the cells, we transfected the PANDA plasmid into a cell line that can express an inducible MS2-tagged mRNA (12). The cell line is called E6 and harbors a doxycycline-inducible gene that encodes a globin-CFP coding sequence. The nuclear transcription sites are easily detectable by RNA FISH with a probe to the CFP region. RNA FISH in control cells showed the cytoplasmic PANDA puncta (detected with the PANDA probes) as well as the E6 transcription sites in the nucleus (detected with the CFP probes) (Figure 5A). However, when RNase and ActD were used, the PANDA puncta persisted while the nascent transcripts at the E6 transcription sites disappeared (Figure 5A,B), implying that the PANDA probes are not detecting RNA and that ActD is inhibiting transcription.

### RNA FISH can detect both strands of cytoplasmic plasmid DNA

**Figure 6.**
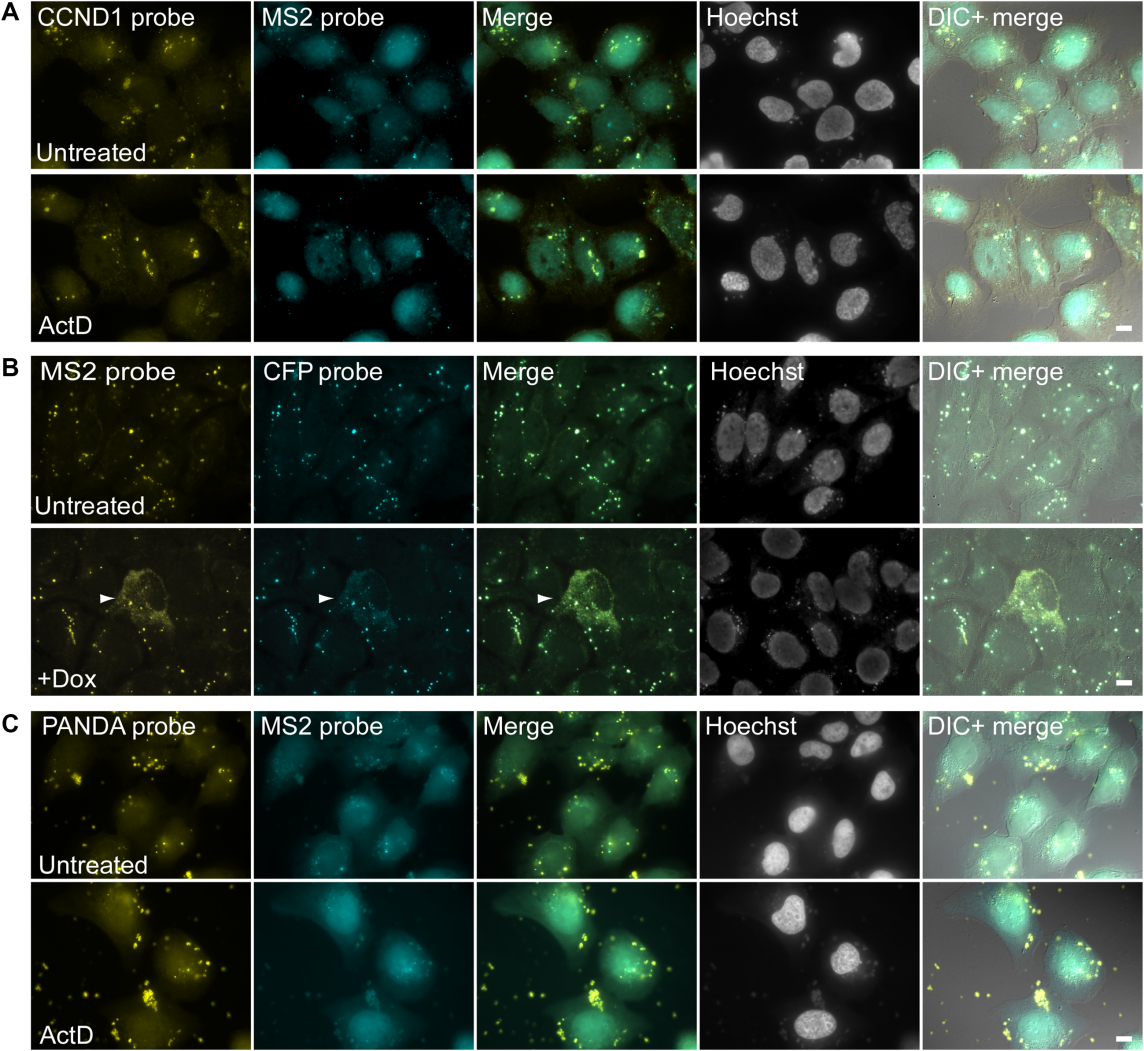
Two different probe sets to the same plasmid colocalize in cytoplasmic puncta detected by RNA FISH. (**A**) U2OS cells were transfected overnight with a plasmid containing the cyclin D1 (*CCND1*) gene and MS2 repeats in the 3′UTR, and no promoter, using PolyJet. RNA FISH was performed with two different probe sets to the cyclin D1 (yellow) and MS2 (cyan) regions. Colocalization between the cytoplasmic puncta can be seen in the merge. Actinomycin D treatment did not abolish the cytoplasmic dots (bottom). (**B**) U2OS Tet-on cells were transfected overnight with a plasmid containing the E6 gene that encodes a CFP fusion protein and has MS2 repeats in the 3′UTR. RNA FISH was performed with two different probe sets to the MS2 (yellow) and CFP (cyan) regions. Colocalization between the cytoplasmic puncta can be seen in the merge. The detection of the cytoplasmic puncta was not affected when the E6 mRNA was transcribed after induction by doxycycline (dox). Arrowheads point to a cell containing E6 mRNA. (**C**) U2OS cells were transfected overnight with a plasmid containing the PANDA gene on the antisense strand and the cyclin D1 gene and MS2 sequences on the sense strand. There was colocalization between the cytoplasmic puncta detected with the PANDA (yellow) and MS2 (cyan) FISH probes. Actinomycin D treatment did not abolish the cytoplasmic dots (bottom). Hoechst DNA counterstain is in cyan. Scale bars = 10 μm.

To validate that different RNA FISH probe sets can recognize different regions of the same plasmid, cells were transfected with the cyclin D1 plasmid that includes MS2 sequences in its 3′UTR (and does not have a promoter). The RNA FISH protocol was performed with probe sets against the CCND1 and MS2 regions and colocalization between the signals was observed (Figure 6A). These signals did not disappear when transcription was inhibited by actinomycin D (Figure 6A). In addition, we transfected U2OS Tet-on cells with the Tet-inducible plasmid called E6 (same as in the cells stably expressing the E6 gene, as used in Figure 5) that also has MS2 sequences in the 3′UTR and encodes a CFP fusion protein (12), which was then detected by probe sets against the CFP and MS2 regions. Here too, there was colocalization of the signals (Figure 6B). Notably, for the E6 gene, when it was induced to transcribe by the addition of doxycycline to the medium, then the E6 mRNA could be detected with the MS2 probe demonstrating the difference between the real RNA FISH signal (small cytoplasmic RNA dots for single mRNA molecules) versus the more large cytoplasmic DNA puncta (Figure 6B bottom). Finally, the colocalization of two different probe sets was also observed when each probe set was targeted to one of the DNA strands. We generated a plasmid that contains the PANDA lncRNA sequence on the antisense strand while the cyclin D1 gene and MS2 sites were on the opposite sense strand. Probes to PANDA and probes to MS2 colocalized in the same cytoplasmic dots (Figure 6C). Altogether, these data showed that both strands and different regions of the plasmid DNA are available for hybridization with the RNA FISH probe sets.

### Cytoplasmic DNA is the molecule that can be detected by RNA FISH

**Figure 7.**
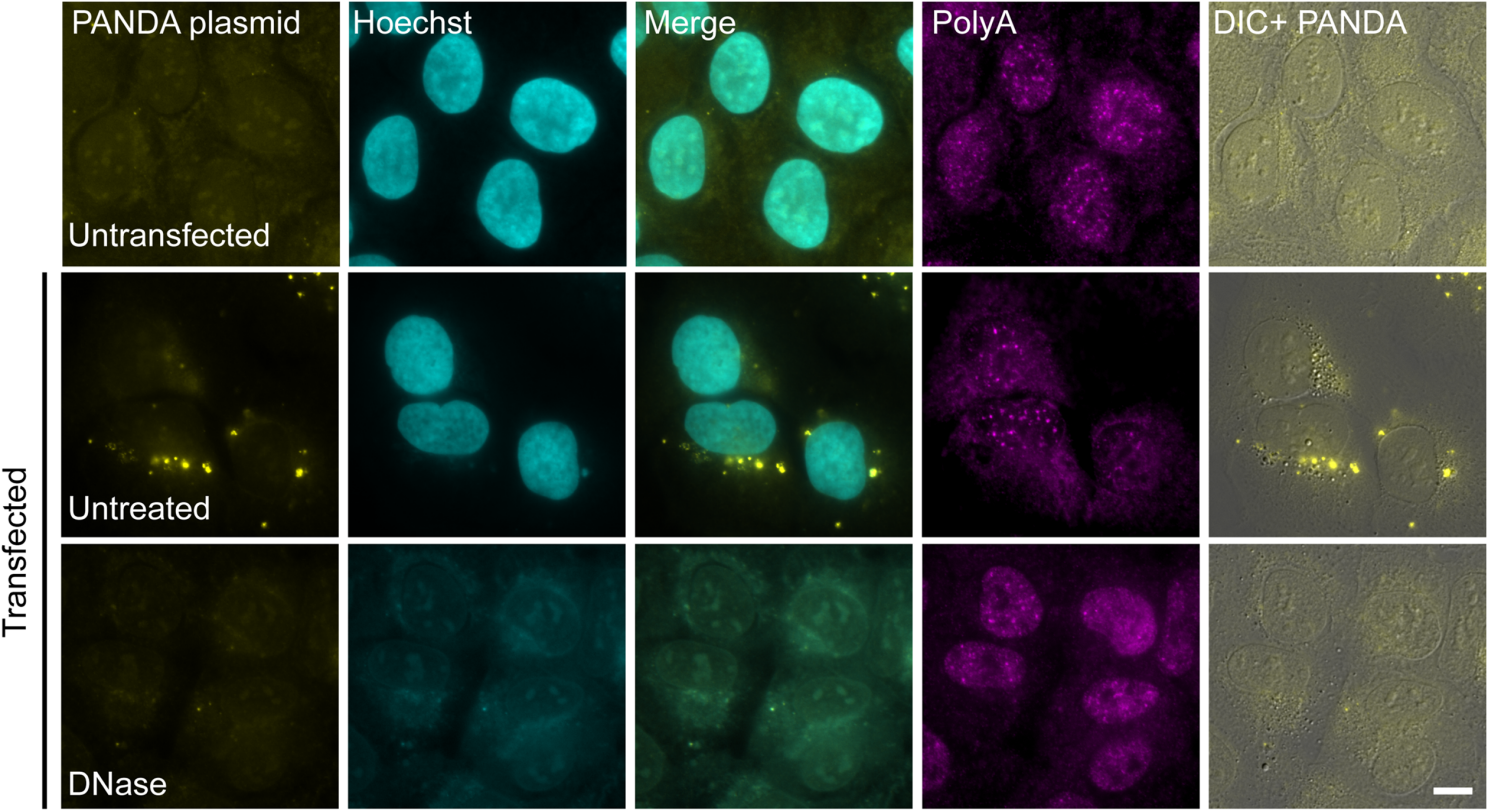
DNase treatment abolished cytoplasmic puncta detected by RNA FISH. U2OS cells transiently transfected overnight by PolyJet with the PANDA plasmid before (middle) and after (bottom) DNase treatment. RNA FISH was performed with probes to PANDA (yellow) and an oligo-dT probe to detect poly(A)+ RNA (magenta). DNase abolished the PANDA and Hoechst signals, but did not affect the poly(A) signal. Hoechst DNA counterstain is in cyan. DIC is in gray. Scale bar = 10 μm.

**Figure 8.**
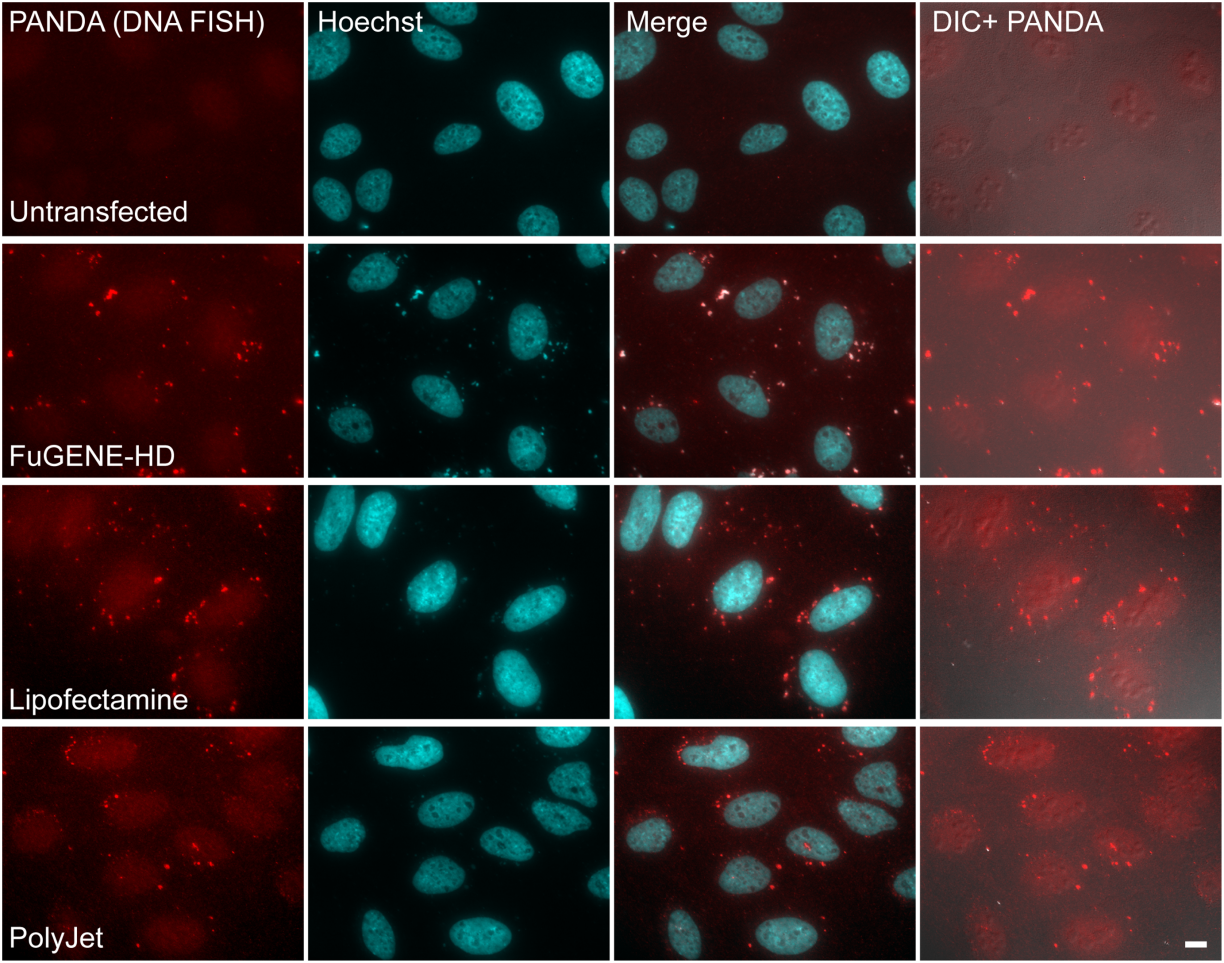
The cytoplasmic puncta forming after transient transfection are detected by DNA FISH. Cytoplasmic puncta (red) in U2OS cells are detected after transfection by a DNA FISH protocol including a denaturation step, with a probe against the PANDA sequence. Cells were transfected overnight with the PANDA plasmid using different transfection reagents. Hoechst DNA counterstain is in cyan. DIC is in gray. Scale bar = 10 μm.

Since these experiments suggested that plasmid DNA was being detected in the cytoplasm by probe sets containing 20-mer probes and no DNA denaturation step to open the DNA double helix, we treated cells that were transiently transfected with the PANDA plasmid, with DNase, in order to remove DNA from the cells. In this case, the cytoplasmic puncta and the nuclear DNA staining disappeared, whereas poly(A)+ RNA signal in the cells (observed with a fluorescent oligo-dT probe) remained intact (Figure 7). The same results were obtained when we treated the cells with DNase after transient transfection with the three plasmids that containing the sequences of CCND1, MEG3 and MS2 (Supplementary Figure S5). Finally, we showed using a DNA FISH protocol that includes a denaturation step, that plasmid DNA appears as cytoplasmic dots following transient transfection (Figure 8).

Altogether, these results demonstrate that plasmid DNA can be detected in the cytoplasm with RNA FISH probes. Since no denaturation step is required, we can speculate that the DNA is not yet packaged right after transfection, and that the FISH protocol is capable of opening the double stranded DNA in the cytoplasm. Moreover, the opening of the strands in the cytoplasm must be stable enough so that there is no competition of the complementary DNA strand with the FISH probes. Only nucleofection that is supposed to directly deliver the plasmids into the nucleus after transfection showed some nuclear signal under these conditions. It has been shown that plasmid DNA gradually changes its nucleosome packaging profile over time after transfection (20) and future experiments will be able to determine the dynamics of the packaging of exogenous DNA. The finding that DNA can be detected by RNA FISH without a denaturation step should bring caution when performing RNA FISH experiments under conditions of transient transfections, especially when counting of RNA molecules in the cytoplasm is to be obtained. Finally, these findings can be of use for the future study of DNA translocation into the nucleus and its packaging after transfection, and possibly for the detection of traces of DNA in the cytoplasm of cells, as has recently been reported for some diseased states (21,22).

## Supplementary Material

gkz645_Supplemental_File
