# Supplementary material for: Cytoplasmic DNA can be detected by RNA fluorescence in situ hybridization
Source: Nucleic Acids Res. 2019 Jul 24;47(18):e109. doi: 10.1093/nar/gkz645 (PMC6765201; doi:10.1093/nar/gkz645)
Supplement: gkz645_Supplemental_File [file gkz645_supplemental_file.pdf]

Supplemental figures

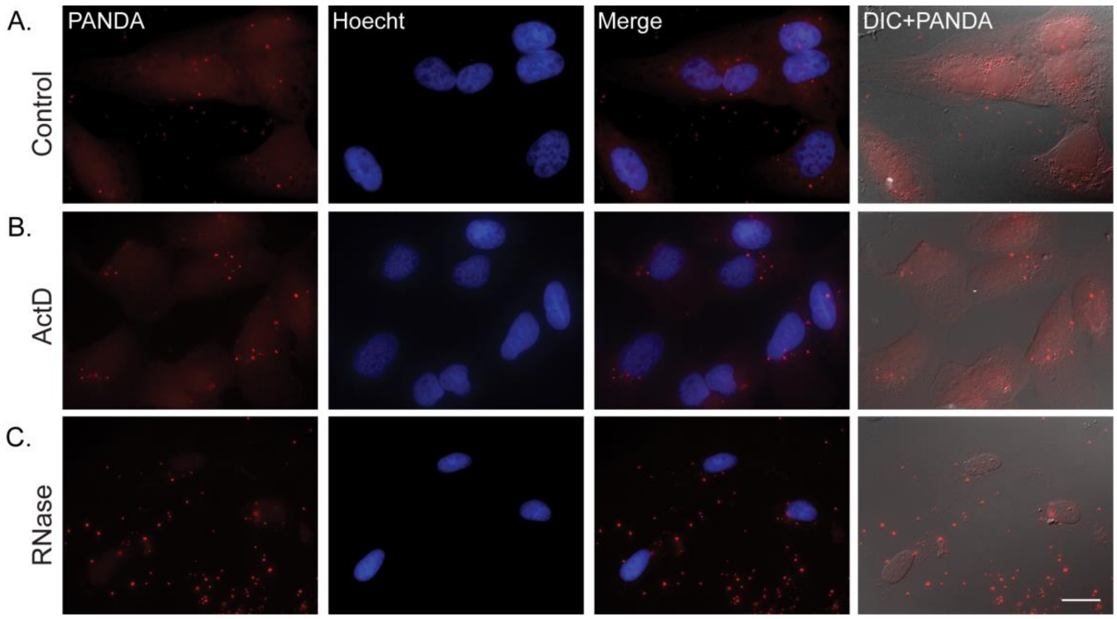

Figure S1. PANDA plasmid can be detected by RNA FISH. (**A**) U2OS cells were transiently transfected overnight by PolyJet with a construct that contains the PANDA gene with a promoter. Untreated cells and cells treated with actinomycin D (2 hrs) or RNase all showed cytoplasmic puncta with a FISH probe set to PANDA (red) compared to untransfected cells. Hoechst DNA counterstain is in blue. DIC is in grey. Scale bar = 10 µm.

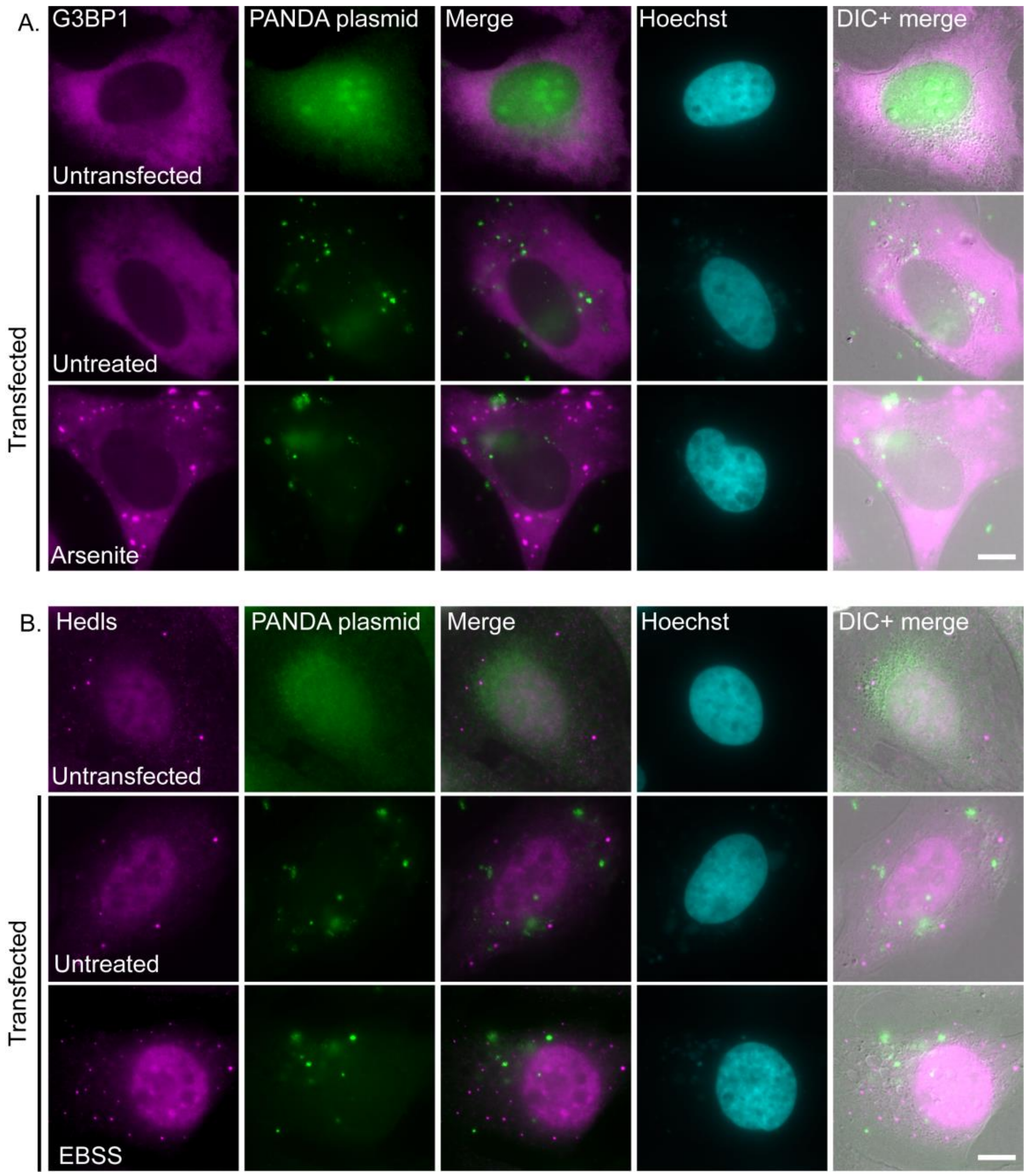

Figure S2. Cytoplasmic DNA puncta do not colocalize with P bodies or stress granules. U2OS cells were transiently transfected by PolyJet with the PANDA plasmid and then underwent the RNA FISH protocol with probes to PANDA (green). Cells were then stained by immunofluorescence (magenta) with an antibody to (**A**) G3BP1 that detects stress granules only after their induction by arsenite (45 min), or to (**B**) Hedls, a P body marker that detects P bodies under normal conditions, and under

conditions of amino acid starvation (EBSS medium) that increase P body numbers. No colocalization between the DNA puncta and P bodies or stress granules was observed. Hoechst DNA counterstain is in cyan. DIC is in grey. Scale bar = 10 µm.

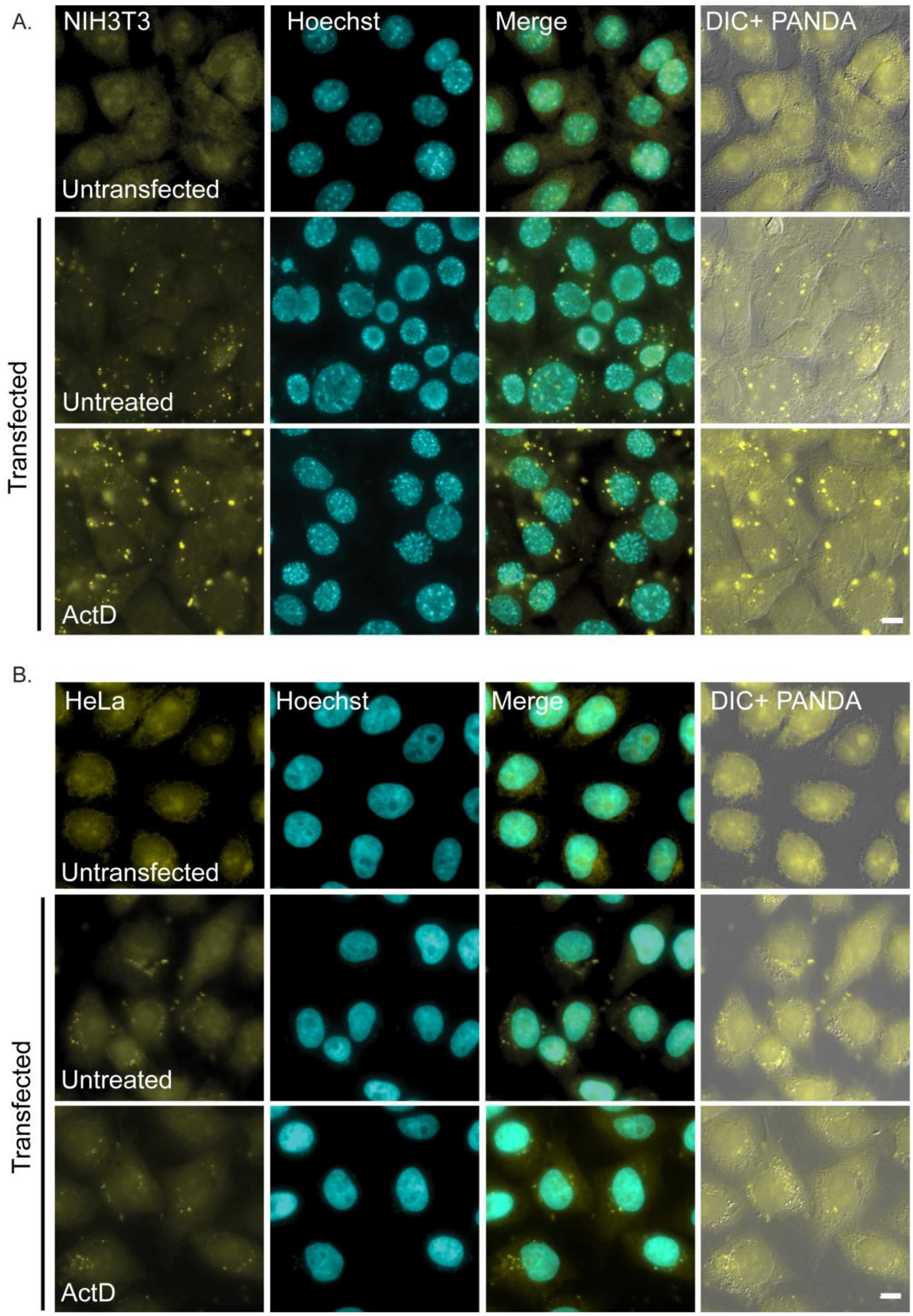

Figure S3. Plasmid DNA can be detected by RNA FISH in different cell types. (**A**) Mouse NIH3T3 cells and (**B**) human HeLa cells were transiently transfected with a

construct that contains the PANDA gene. After transfection and RNA FISH with a probe set to PANDA (yellow), both cell types showed cytoplasmic puncta before (middle) and after (bottom) actinomycin D treatment, in contrast to untransfected cells that did not show any cytoplasmic dots. Hoechst DNA counterstain is in cyan. DIC is in grey. Scale bar = 10 µm.

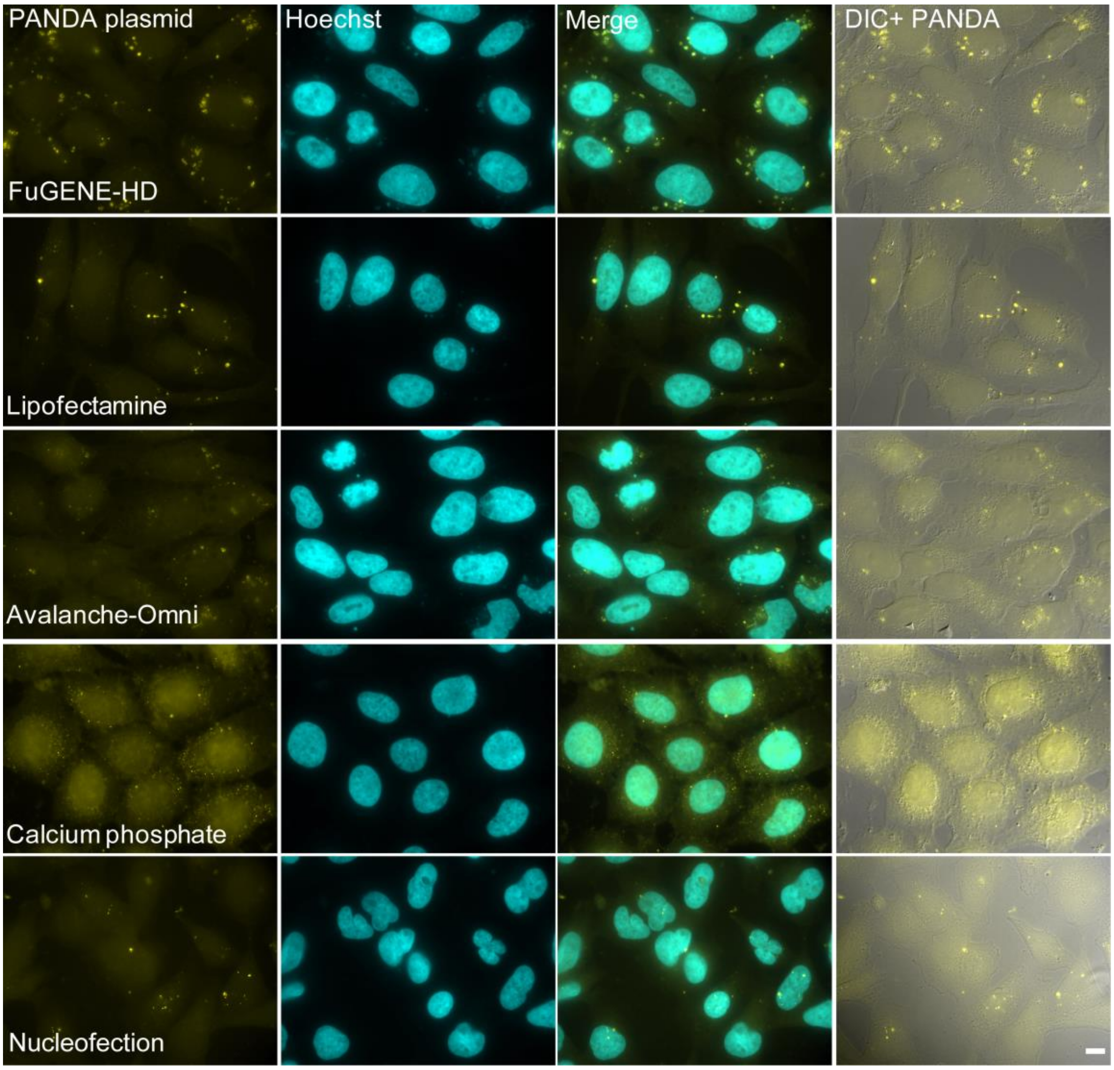

Figure S4. Plasmid DNA can be detected by RNA FISH using a variety of transfection procedures. U2OS cells were transiently transfected with a construct that contains the PANDA gene. RNA FISH with a probe set to PANDA (yellow) detected cytoplasmic puncta with all transfection conditions: FuGENE-HD, Lipofectamine, Avalanch-Omni, calcium phosphate and nucleofection. Hoechst DNA counterstain is in cyan. DIC is in grey. Scale bar = 10 μm.

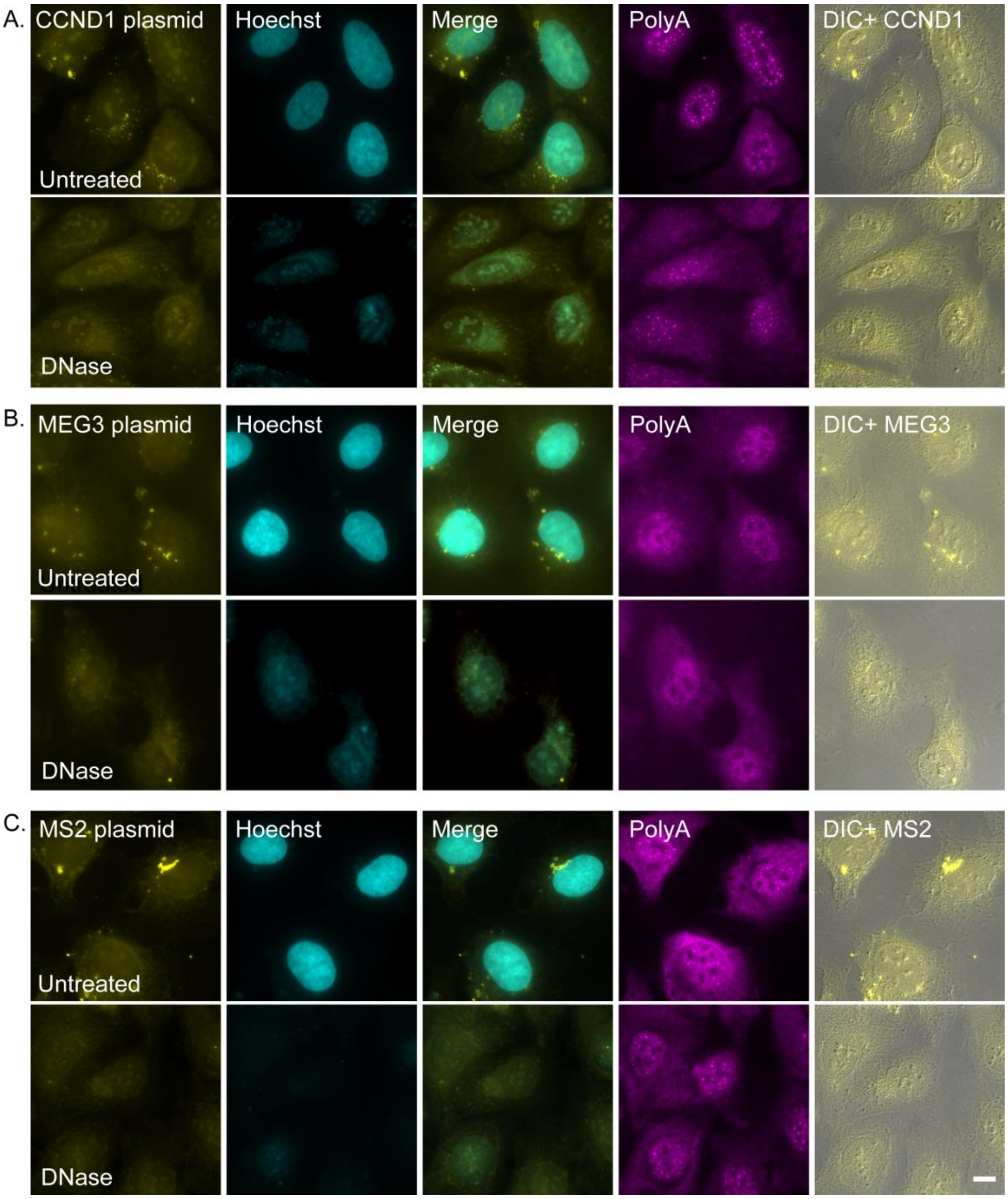

Figure S5. DNase treatment abolished the cytoplasmic puncta detected by RNA FISH. U2OS cells transfected with (**A**) cyclin D1 (*CCND1*), (**B**) MEG3 and (**C**) MS2 plasmids were treated with DNase. DNA cytoplasmic dots detected by RNA FISH (yellow) and by Hoechst (cyan) were abolished after DNase treatment. Detection of poly(A)+ RNA (magenta) was not affected by DNase treatment. DIC is in grey. Scale bar = 10 µm.
